# Visual Measurements of Breathing Parameters in Children With a Particular Focus on Phase Angle: A Pilot Study

**DOI:** 10.7759/cureus.77297

**Published:** 2025-01-11

**Authors:** Israel Amirav, Alon Zvirin, Sapir V Levi, Neta Rabin, Yaron Honen, Or Marudi, Daphna Vilozni, Moran Lavie, Ron Kimmel

**Affiliations:** 1 Department of Pediatrics, Ichilov Hospital, Tel Aviv, ISR; 2 Department of Computer Science, Technion - Israel Institute of Technology, Haifa, ISR; 3 Department of Pediatrics, Montefiore Medical Center, Wakefield Campus, Bronx, USA; 4 Department of Pediatric Pulmonology, Tel Aviv Sourasky Medical Center, Tel Aviv, ISR; 5 Department of Electrical and Computer Engineering, Technion - Israel Institute of Technology, Haifa, ISR

**Keywords:** phase angle, phase shift, remote monitoring, respiratory rate, tidal volume

## Abstract

Introduction

Pediatric respiratory monitoring, crucial for assessing children's health, particularly those with respiratory diseases, often relies on invasive or cumbersome methods. Here, we propose a non-invasive approach using a video depth camera to measure breathing parameters in children, offering innovation and promise.

Aims

We aim to introduce and validate a straightforward remote procedure for measuring crucial breathing parameters in children. These include respiratory rate (RR), volumetric changes during inhalation and exhalation, and the phase angle (PA) between chest and abdomen expansions.

Methods

The proposed method involves detecting three feature points - nipples and navel - using a video depth camera. A 30- to 60-second video is recorded to track chest and abdomen movements. Analysis of feature point locations, distances between them, and signal frequencies is conducted to estimate respiratory parameters. To validate the accuracy of our method, we employed mechanical lung simulators within dolls for procedure testing and measurement accuracy verification. Additionally, recordings of pediatric patients, both healthy and with respiratory diseases, were analyzed to correlate computational parameter estimations with physician assessments, ensuring the reliability and effectiveness of our approach.

Results

Our results show a strong correlation between simulator inputs and algorithm estimations, validating our method's accuracy. Additionally, applying the procedure to pediatric patient recordings significantly correlates with physician assessments, notably, marking the first remote measurement of the respiratory PA.

Conclusions

This remote procedure presents a promising alternative for pediatric respiratory monitoring, offering accurate measurements without invasive techniques or extensive equipment. The robust correlation between computational estimations and physician assessments underscores its reliability, suggesting potential for broader clinical applications and advancements in pediatric respiratory care.

## Introduction

Analysis of breath motion patterns and pulmonary function testing are essential for assessing the respiratory functioning of children. Online automatic detection can assist physicians and pediatricians in hospitals, clinics, and even field conditions, especially when one has a real-time, non-contact device for capturing and analyzing relevant information. Conventional medical technologies in this context usually employ sophisticated and costly devices that generally require patient cooperation, patient contact, and expert manual operation. Moreover, these technologies (e.g., spirometers) cannot be used in non-cooperative infants and/or young children.

There is an increased need for automatic or semi-automatic, low-cost, user-friendly, objective systems that can perform as well as traditional technologies. In the wake of the COVID-19 pandemic, the demand for remote, non-touch detection and analysis systems has significantly increased.

Here, we introduce a simple and non-invasive technology that can overcome the above-mentioned concerns, particularly for infants and young children with respiratory problems. We focus on three parameters characteristic of normal and abnormal breathing: respiratory rate (RR), volumetric estimation of chest expansion, and phase angle (PA). The most basic parameter is the RR. RR plays an important role in routine clinical assessment for disease diagnosis, prognosis, and treatment of many clinical conditions. Several studies indicate that normal RR is the best individual finding for ruling out pneumonia [1,2], and the RR has been suggested as the most sensitive in the detection of any clinical deterioration [3]. Although normal RR ranges, according to age, appear frequently in the literature [4,5], there is still a need for an accurate and consistent method of measuring it, and even more so of relating this measurement to a valid clinical diagnosis of healthy vs. abnormal pulmonary functioning [6-8].

Phase shift, also termed PA in respiration, is the temporal offset of the same frequency between the rib cage and abdomen movements. Expansion of the rib cage and abdomen occurs in near synchrony in normal tidal breathing in children, and asynchronous breathing is a clinical parameter that could indicate respiratory distress. First introduced by Konno and Mead [9] as a measure of volume displacements between the rib cage and abdomen, it was later suggested as an indicator of upper airway obstruction [10,11]. The PA is generally considered an important factor in assessing various pulmonary functionalities [12,13], specifically in infants [14-16]. Traditionally, this parameter is measured by a double-belt device placed on the rib cage and the abdomen - a method known as respiratory inductance plethysmography (RIP) [17-19]. No previous study has shown the ability to measure PA remotely. Here, we explored the hypothesis that this parameter can now be measured by tracking feature point locations as observed from a remote camera’s point of view.

Tidal volume (TV) is a breathing parameter traditionally measured by spirometers. Changes in TV can indicate normal vs. restrictive or obstructive ventilation. Previous studies indicate that even when measuring TV using computed tomography (CT) images in diagnosing healthy or lung-injured patients [20], and when utilizing mechanical ventilators in cases of respiratory distress [21], calibration with a respirometer is necessary for accurate volumetric estimations. Since spirometers require a certain degree of patient cooperation, non-invasive optoelectronic plethysmography methods for measuring respiratory volume changes are an evolving field of research [22-24].

The primary objective of this report is to describe the development of a low-cost camera-based portable system for remote measurements of RR, PA, and volumetric estimation of breathing. Importantly, we also aimed to verify the performance of this system using baby doll lung simulators. Our last objective was to check the system's applicability to humans through a pilot study in children.

## Materials and methods

General

Data were collected at the Pediatric Pulmonary Unit of Dana-Dwek Children’s Hospital in the Tel Aviv Sourasky Medical Center, Tel Aviv, Israel. We will first describe the devices employed in this study - the camera and lung simulators. We will then describe the experimental settings and complete the methods section by describing the data's preprocessing, collection, analysis, and verification using baby doll lung simulators.

Camera

The current technology is based on the Intel (Intel Corporation, Santa Clara, CA, USA) RealSense D435 depth camera [25]. This low-cost (∼150$) portable camera can capture and stream depth data of moving objects. As such, we selected this camera to capture chest motions during breathing. The camera is programmed with vision processors, depth, and tracking modules, and is backed by an open-source multiplatform software development kit (SDK) called librealsense. It includes an InfraRed (IR) projector, two IR cameras on the left and right, and a color camera. The IR projector allows the camera to detect depth by emitting a static IR pattern on the scene, and the two IR cameras send raw data about the scene to the vision processor. This vision processor determines the depth values by calculating the distance from the camera to the pixels in both the left and right cameras. The Intel D435 depth camera captured 30-second videos consisting of color and depth frames at ∼15 frames per second and 480 × 640 pixel resolution. The color and depth frames are pixel-wise aligned, allowing a direct mapping of 2D pixel coordinates to real-world 3D coordinates in mm. The data was uploaded to a computer and analyzed using RealSense analysis software (Intel Corporation).

Lung simulators

RR, PA, and TV were estimated on video recordings of two custom-designed dolls with a programmable lung simulator, a baby-sized and an adult-sized doll. These two simulators have different designs. The baby doll is capable of accurate pre-determined (programmable) RR and PA input, whereas the adult doll can determine RR and TV input. The manufacturer confirmed the accuracy of RR and PA input values to the baby doll [26]. RR and TV input values for the adult doll were determined through a programmable ventilator. Three colored circular stickers were placed on the dolls’ bodies to detect the feature points, substituting the nipples and navel easily.

Experiment setup

Several predetermined configurations of breathing parameters of interest (RR, PA) were tested. Tested configurations of the baby doll consisted of RRs of 20, 30, 40, 50, 60, 70, and 80 breaths per minute (BPM) and PAs of 0, 30, 60, 90, 120, 150, and 180 degrees. Tested configurations of the adult doll consisted of RRs of 10, 25, and 40 BPM and TVs of 100, 150, and 200 mm. All recordings followed a rigid filming protocol, ensuring the doll was lying face up, the camera positioned 40 to 50 cm above and facing the doll, capturing the upper part of the body in a frontal view. Three colored circular stickers were placed on the dolls’ bodies to detect the feature points, substituting the nipples and navel easily.

Calculations of the RR, PA, and TV were performed on sequential sliding windows of 15 seconds. In other words, at each point in time t, during the video recording, these parameters were evaluated on a sequential set of frames accumulated during the time interval (t-15, t), consisting of the last 15 seconds and up to the present moment. If a single scalar value was required, representing a result obtained from the whole recording, we extracted the most occurring value amongst all sliding windows (RR), or an average or median value (for PA and TV parameters).

**Figure 1:**
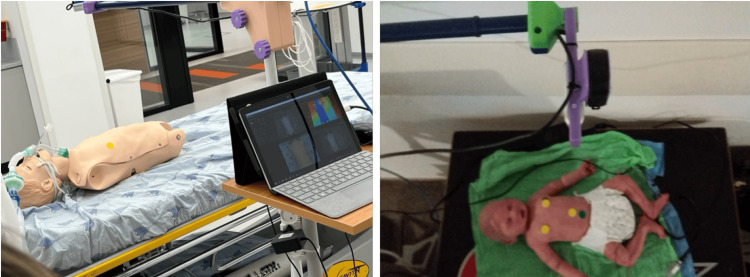
System setup displaying doll simulators, adult (left) and baby (right). The dolls are lying face up, the camera 40 to 50 cm above them, and capturing a frontal view.

The key feature locations in 3D also determined the PA between chest and abdomen motion, which was calculated on a 15-second sliding window. The PA was calculated separately between feature point locations (nipples and navel) and between relative motions of the virtual belts (stripes covering the chest and abdomen). The experiment setup is displayed in Figure 1. Preliminary testing showed that a 2D video was enough for accurate RR calculation, whereas 3D information was better suited for PA and volumetric measurements.

Data preprocessing

**Figure 2:**
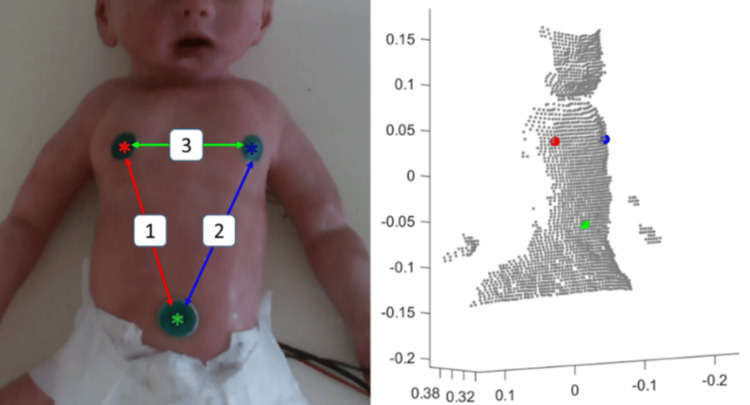
Baby doll simulator with detected markers on nipples and navel, and distances between markers. Color image (RGB, left) and corresponding 3D point cloud (right). The 3D model is rotated and down-sampled for visualization. RGB, Red, Green, Blue

First, each video frame was processed separately, keeping its timestamp. The stickers were detected, centroids extracted, and distances between them calculated. The distances were then normalized and plotted as a function of time. By applying the Fourier transform (time to frequency domain), the prominent frequency corresponds to the RR (BPM). The stickers were detected by a color threshold on the original RGB (Red, Green, Blue color) image or on a color transformation to HSV (Hue, Saturation, Value) space. Ordering the sticker locations is possible, assuming the patient is photographed upright, the chest appearing in the upper part of the frame, and the abdomen in the lower part. We tested several configurations for the sticker locations to find which distances between them, and more specifically the fluctuations of these distances over time, best capture the body motion corresponding to breath cycles. Results show that optimal locations are indeed the two nipples and navel. Using aligned color and 3D frames from the depth camera, obtaining the 3D spatial coordinates corresponding to each pixel in the color frame is straightforward. Since the 3D data is somewhat noisy and limited in accuracy, smoothing the 3D coordinates by averaging the coordinates of a small neighborhood of pixels proved to yield better performance. Figure 2 displays detected sticker centroids and the corresponding 3D coordinates. We applied a sliding window of 15 seconds to check whether the breathing pattern was consistent over time.

Respiratory rate

**Figure 3:**
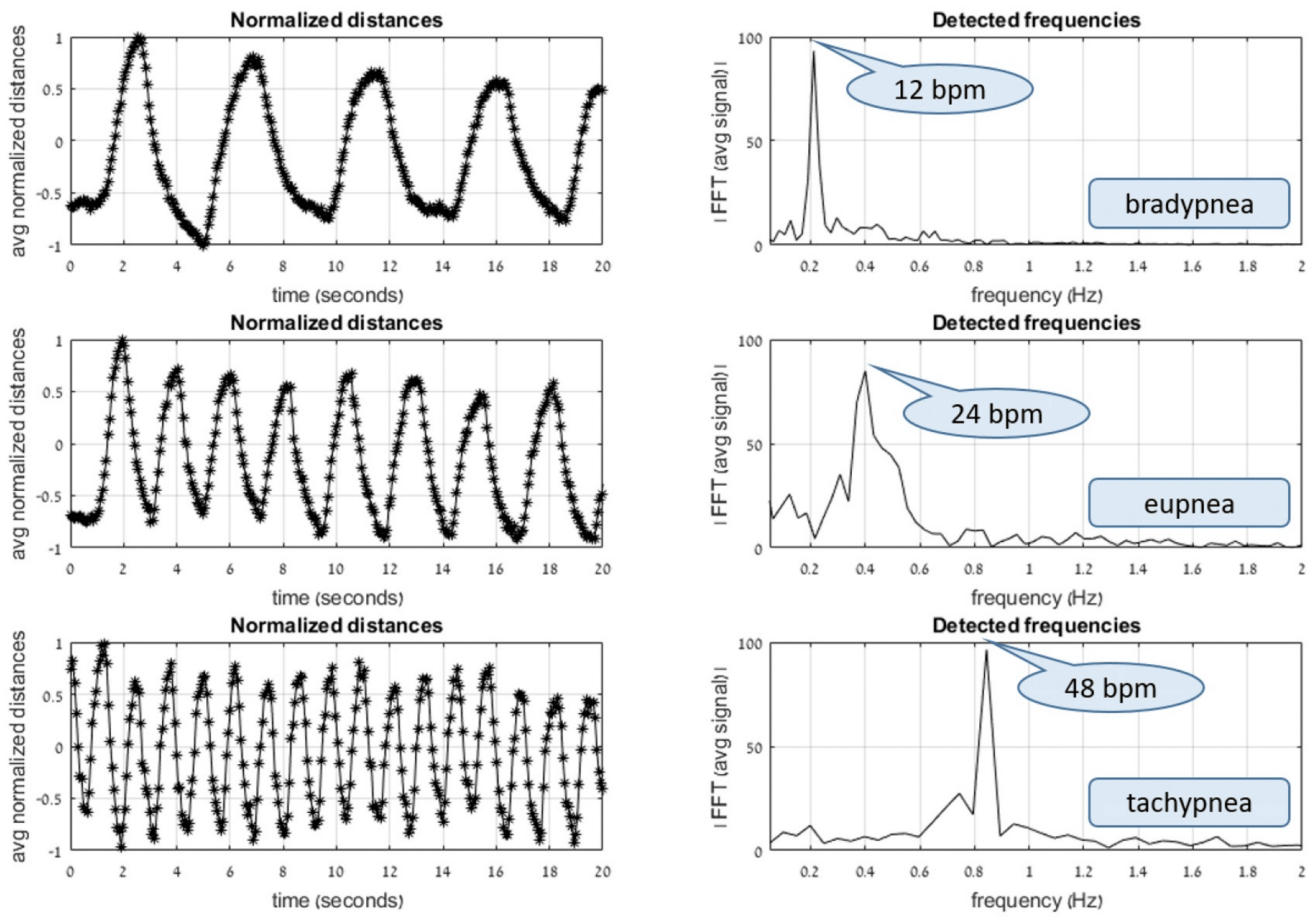
Example demonstrating analysis of slow, medium, and fast breathing (top, middle, and bottom, respectively). The left graphs plot the averaged normalized distances between feature locations as a function of time. The right graphs are the corresponding Fourier transforms of these signals. When the breathing is fairly consistent over time, there is one prominent value observed in the frequency domain, corresponding to the respiratory rate.

Following the distances between feature points as a function of time allows us to track the breath cycle. The RR, commonly measured by BPM, can be estimated by counting the number of cycles or peaks in the signal, or the number of zero-crossings when the signal’s amplitude is normalized in the range (-1, 1). Applied to sinusoidal waves, this approach works fine. Still, in practice, motion is not smooth, and the signals contain artifacts due to camera noise, limitations of depth accuracy, and small body perturbations separate from respiratory motion resulting from breath cycles. Therefore, we used a Fourier transformation approach, transforming the time-based signal to the frequency domain and obtaining the dominant frequency, thus determining the RR. Figure 3 shows the distance functions and their Fourier transforms when applied to a human adult requested to enact slow, medium, and fast breathing patterns, simulating bradypnea, eupnea, and tachypnea.

Phase angle

**Figure 4:**
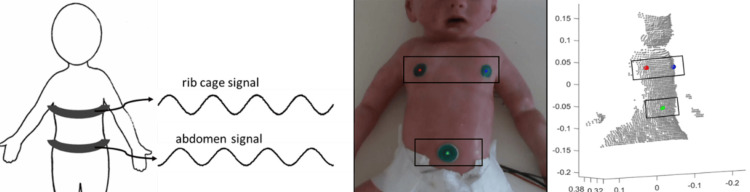
Respiratory inductance plethysmography (RIP) double-belt device. Schematic depiction (left), following the description in the seminal paper by Sinton and Suntheralingam [19], and virtual belts on relevant chest and abdomen regions of interest (middle, right). The area enclosed by these belts remains constant during the video, parallel to the xy plane, but the distances from the camera (the z coordinates) change and correspond to the motion of the upper body.

PA is a measure of the temporal movement of one body compartment in relation to another during each breath, and, in this case, was determined by the offset between the chest and the abdominal compartments. A PA of 0° represents complete synchrony, indicating that the two compartments move together during inhalation and exhalation. A PA of ±90° would represent complete asynchrony, whereas 180° represents paradoxical breathing, according to the terminology used by Hammer and Newth [27]. Generally, PA is measured with a RIP double-belt (chest and abdomen) device. Here, the PA was calculated by the movements of 3D locations of the key feature points, nipples, and navel. Two "virtual" belts were defined, approximating the horizontal strips of the RIP belts covering the rib cage and abdomen, enabling estimation of the average. Figure 4 depicts a schematic view of the conventional RIP device and the "virtual" belts.

The calculation of the PA is described in Appendix 1.

Tidal volume

The volume of tidal breath is approximated by Riemann sums over a predetermined region of interest (ROI). We assume the background behind the patient is stationary; therefore, the upper body expansions and contractions are equivalent to the volume between the surface of the upper body and the plane beneath the body. Each camera pixel has its own x, y, and z coordinates, thus enabling the computation of a Riemann sum on a rectangular grid. We assumed that the grid coordinates on the xy plane remain constant during the video filming, and thus, the changes in depth (derived from changes in the z coordinate) accumulate to the volume.

## Results

Estimating the RR by 2D (pixel) distances was practically the same as estimation by 3D (mm) distances. In practice, distances in 2D were found satisfactory enough for RR estimation, but 3D has the benefit of allowing phase angle and volumetric measurements.

**Figure 5:**
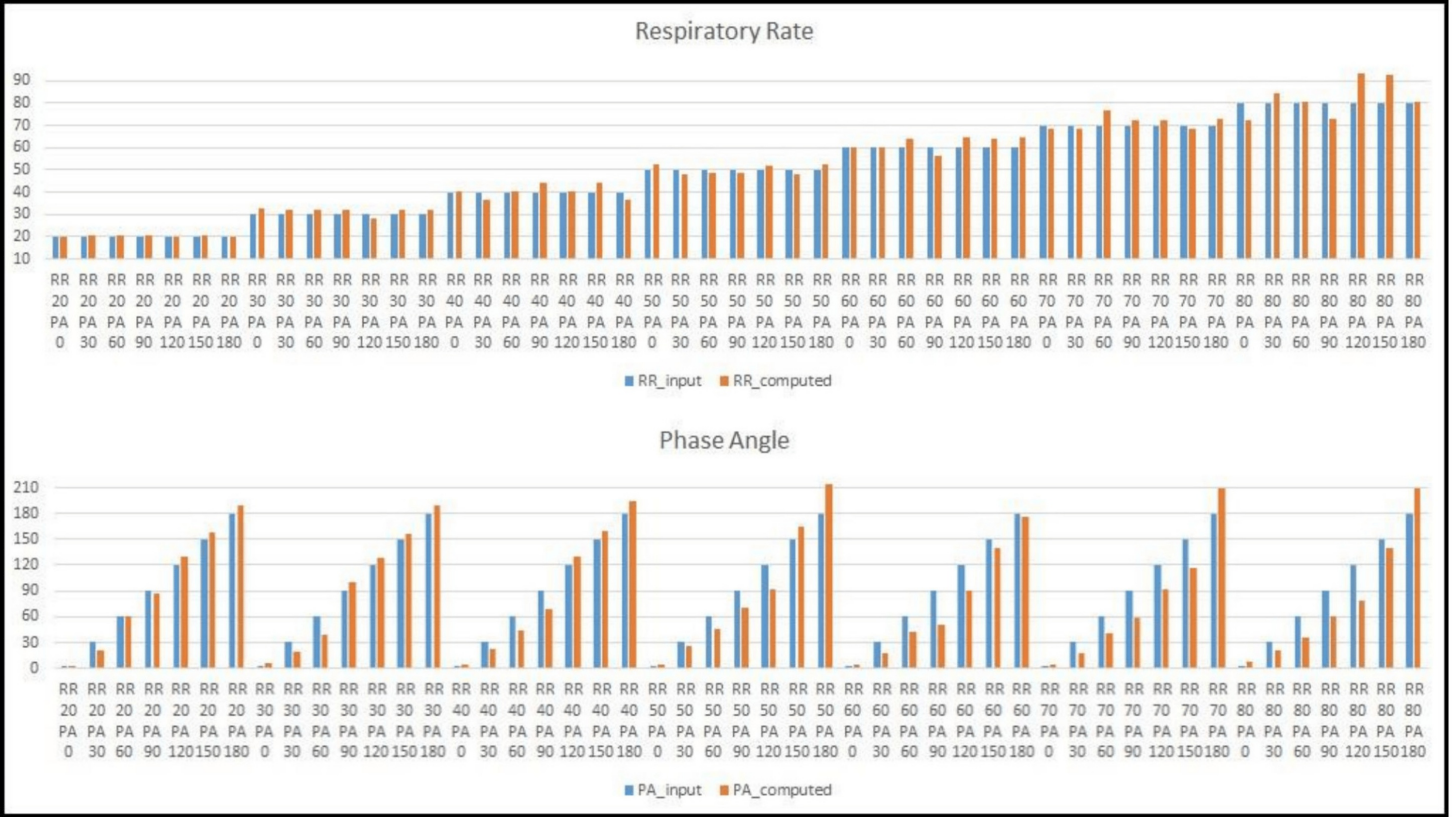
Respiratory rate (top) and phase angle measurements (bottom) on the baby doll. The blue color indicates simulator input, and the orange color indicates calculated values. The calculated RR and PA values present a close approximation of the programmed input to the simulator. PA, Phase angle; RR, Respiratory rate

**Figure 6:**
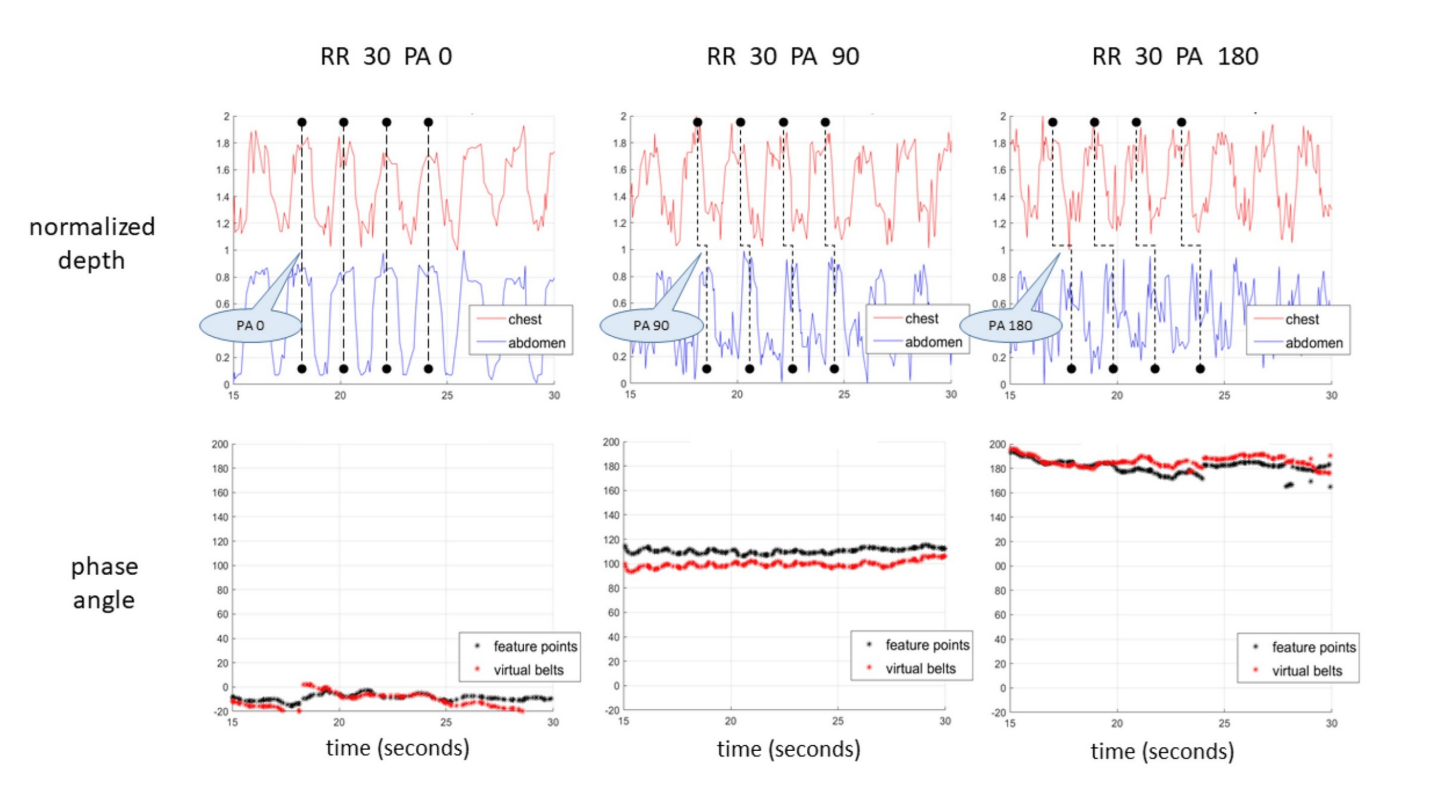
Phase shift analysis of three tested configurations. PAs of 0° (left), 90° (middle), and 180° (right), all with a respiratory rate of 30 BPM. The top graphs display the chest and abdomen motion (red and blue, respectively) and the corresponding shift in their respective peaks during a few breath cycles. The bottom graphs display the calculated PA values, with calculations performed each time on a sliding window containing the last 15 seconds. Two methods were used to calculate the PA: feature point location (black plot) and virtual belt motion (red plot). PA, Phase angle; RR, Respiratory rate

Figure 5 presents calculated RR and PA values (from the computational algorithm) and the simulated doll parameters. There was a strong consistency between the calculated and the pre-programmed values for both parameters in the various breathing configurations. Figure 6 displays samples of the measured PA on the baby doll, focusing on the relation between the shift in time-based feature location signals and the estimated PAs. A special note is made regarding the PA’s cyclic nature and sign. We follow the conventional “double-argument” arctan formulation, with a range of -180°, 180°. A positive value (in the range 0°, 180°) refers to cases in which the chest precedes the abdomen, and a negative value (in the range -180°, 0°) refers to values in which the abdomen precedes the chest. The baby doll can only be programmed with positive values. In most, but not all, cases of human infants, the PA measurement turned out to be negative, meaning the abdomen slightly precedes the chest.

**Figure 7:**
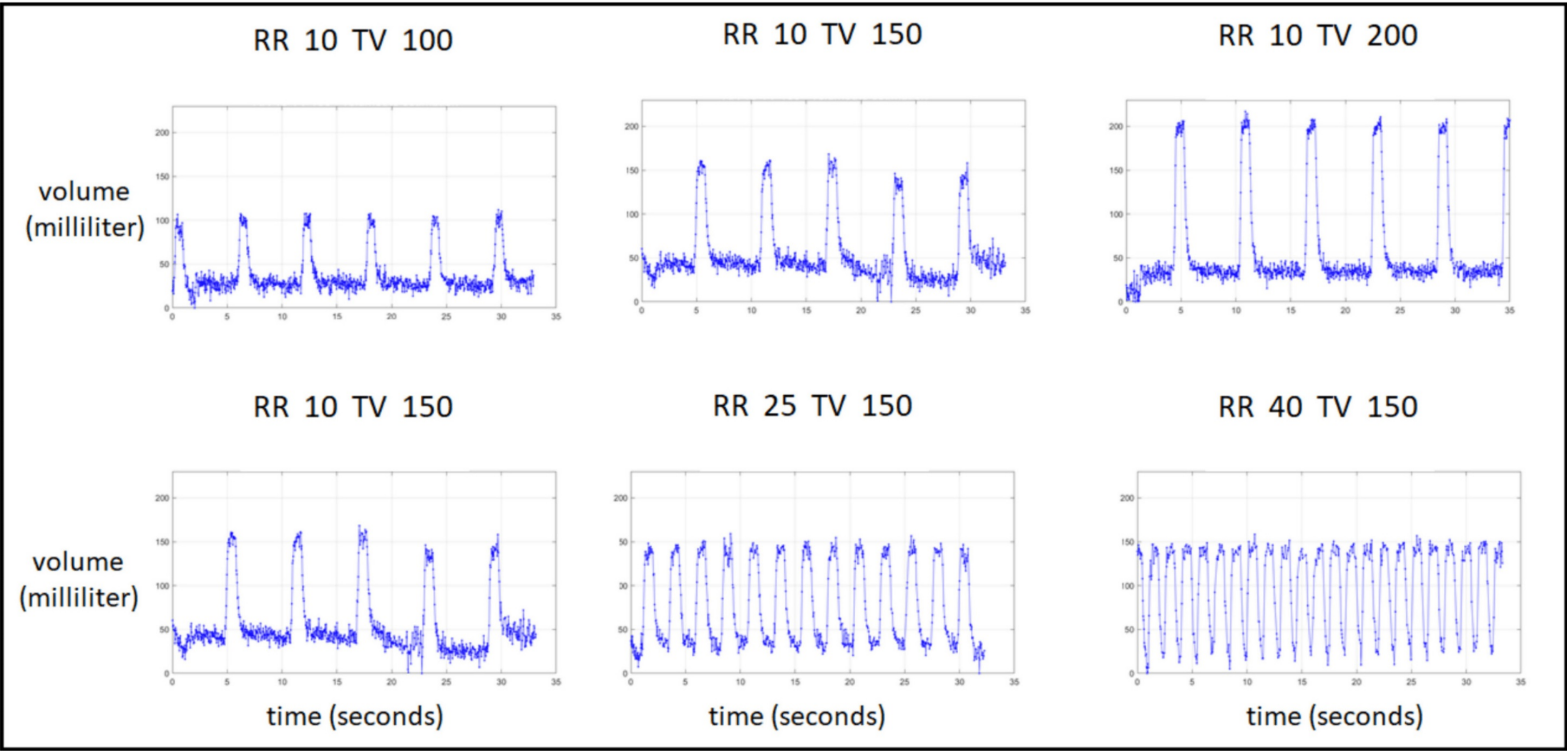
Tracking of tidal volume from videos of the adult doll. The top row depicts configurations with a constant respiratory rate of 10 BPM, and growing tidal volume amplitude inputs of 100, 150, and 200 mm (left to right). The bottom row depicts configurations with a constant tidal volume amplitude of 150 mm, and respiratory rates of 10, 25, and 40 BPM (left to right). RR, Respiratory rate; TV, Tidal volume

**Figure 8:**
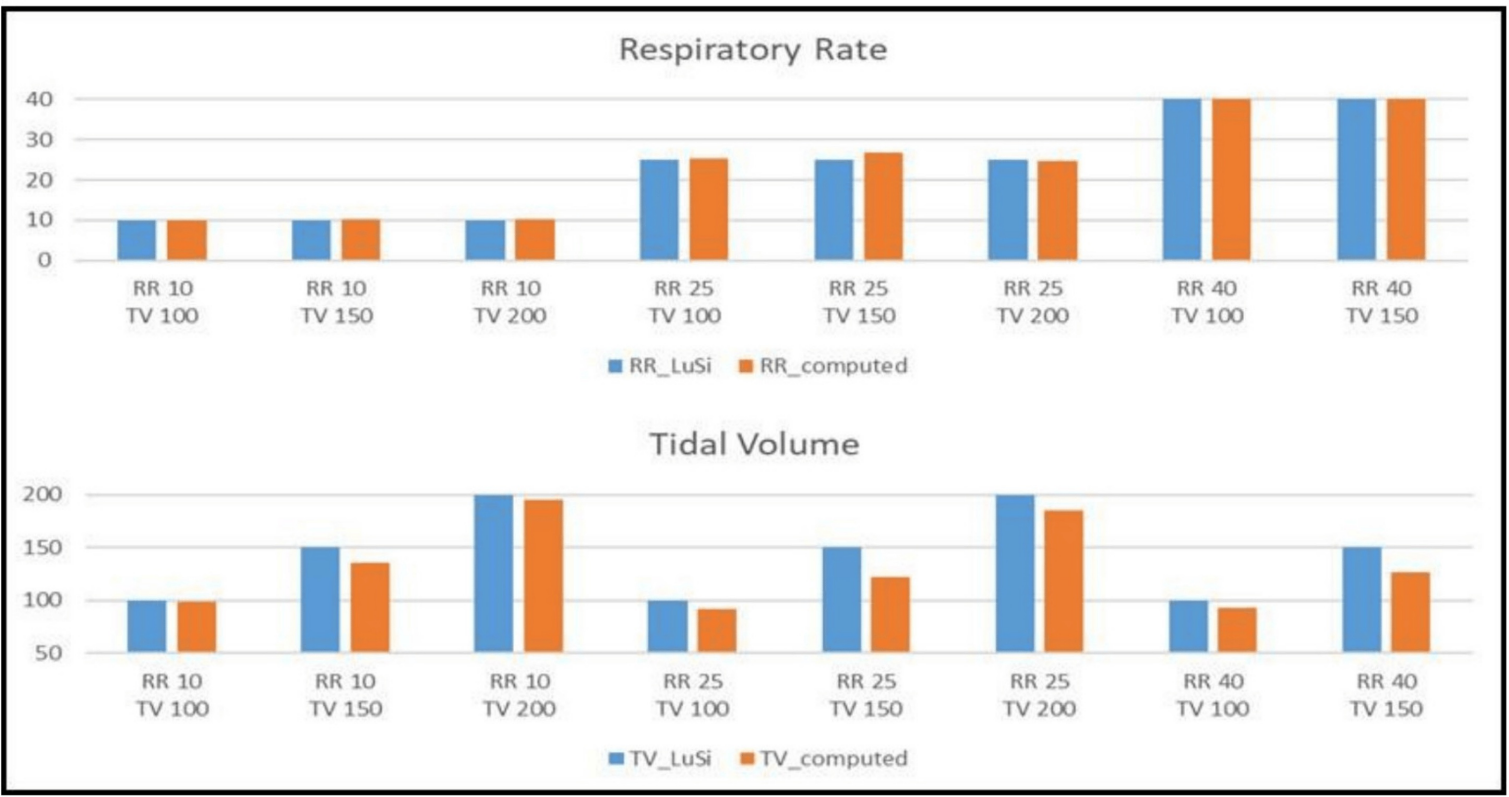
Respiratory rate and tidal volume measurements on the adult doll. RR (top) and TV (bottom) measurements on the adult doll. The blue color indicates simulator input and the orange color calculated values. The calculated RR values present a very close approximation of the programmed input to the simulator. The calculated TV values are slightly smaller than the programmed input to the simulator. RR, Respiratory rate; TV, Tidal volume

TV in the visual recordings was calculated as a Riemann sum of depth coordinates on a fixed xy grid. Sample plots of estimated volume as a function of time (of the adult doll) are displayed in Figure 7. It is immediately apparent that both the cycle period and the amplitude remain constant and consistent during the 30-second video recording. The period closely matches the doll’s input RR, and the amplitude is slightly smaller than that of the doll’s input TV. Figure 8 presents the estimated RR and TV vs. the original input to the simulator on all the tested configurations. In these cases, the BPM was extracted directly from the TV signal, without detection of feature point locations or the distances between them.

Application to humans

A pilot study was performed on children to check the algorithm’s applicability to humans. This study involved both healthy children (a control group) and a subgroup of infants diagnosed with spinal muscular atrophy (SMA) of various ages. To date, 12 patients have participated, following appropriate ethics approval. The study received approval from the TLVMC Helsinki Ethics Committee (approval number 0348-18-TLV) and is registered at the Ministry of Health clinical trials registry (MOH_2019-03-14_006019). Informed written consent was obtained from all participants or their caregivers. For data collection, 30- to 60-second videos of healthy and diseased children were recorded. Patients were lying supine; the camera was 40 to 50 cm above their chest, mounted on a tripod, and connected with a USB cable to a laptop. In this sub-study, we compared RR values between the camera-based method and manual RR measurement by a physician. The PA of the children’s breath was also determined as previously described. The manual RR value was estimated by viewing the recordings and counting breaths during the video playback.

In contrast to mechanical doll simulators, human patients, especially small children, cannot be “programmed” to breathe with preset, value-specific RRs or PAs. As seen in Table 1, the RR results of the automatic procedure closely matched the manual estimation. Furthermore, although this experiment consists of only a small group of children, the results show a clear difference in the measured PA between healthy and SMA patients.

**Table 1:** Comparison of manual and automatic measurements of patients’ RR and PA. RR, Respiratory rate; PA, Phase angle; SMA, Spinal muscular atrophy

Age (year/month)	Gender	Weight (kg)	SMA/control	BPM (manual)	BPM (automatic)	Phase angle (degrees)
1/4	F	7.2	SMA	35	Failed*	-143
3/11	M	15	SMA	24	24.3	-130
9/3	F	21	SMA	22	20.6	-124
9/7	F	23	SMA	28	25.7	-156
2/6	M	12	Control	24	31.6	-105
3/1	F	12	Control	30	32.8	-56
4/3	F	15	Control	16	19.1	-23
4/3	M	19	Control	26	22.5	-39
5/3	M	19	Control	28	28.6	-74
6/11	F	20	Control	14	12.3	-24
8/5	M	36	Control	20	20.8	-27
10/11	M	68	Control	14	13.9	-26
*In this case, the baby constantly moved her hands, thus occluding the stickers on the chest.						

It was not always possible in these patients to achieve a consistent PA measurement over time; we believe this was due to noisy data, as well as the depth accuracy (resolution) of the camera being 0.5 cm, just about the fluctuation range of the key point locations. Smoothing of the data, temporal or spatial filtering, did help in a few cases, as did stretching the sliding window duration to 20 or 30 seconds. A note should be made that the manual measurement in these cases was a single value extracted from the complete video, while the automatic procedure outputs a continuous value depending on the selected duration of the window size. The results of the automatic measurement presented in Table 1 are those obtained from the complete video.

## Discussion

Recent reviews have suggested the potential benefits of a non-contact automatic visual inspection method for respiratory parameters. Firstly, it allows for reduced restriction of patients, avoiding the need for contact with the body and resulting in less pain and discomfort [28]. Secondly, it can improve the quality of care by allowing caregivers to monitor RR, TV, and respiratory patterns without requiring direct patient-clinician contact [29]. Thirdly, it enables continuous and remote monitoring of respiratory vital parameters, providing significant medical information for a continuous patient monitoring system. Additionally, this method can be used in diverse applications, such as sleep studies, sports studies, rehabilitation centers, quarantine centers, and hospital or airport screening during the COVID-19 pandemic [29]. Overall, non-contact automatic visual inspection methods for respiratory parameters offer the potential for improved patient comfort, continuous monitoring, and tailored treatment plans [29].

Several non-contact visual-based methods for estimating breathing parameters, especially RR and TV [30-32], have recently been suggested. Hsu et al. developed a system that uses a personal mobile phone microphone to record the breathing signal and applies the mel-frequency cepstral coefficient and deep neural network to classify exhale, inhale, and silence phases in human breathing behavior [33]. Daqing and Fusang proposed a method that uses a non-contact perception mode to recognize a breath detection area based on the position and distance between sending and receiving devices [34]. Cihui et al. developed a non-contact breath-detecting device that uses light emitted and reflected from the chest or abdomen to obtain a breathing signal [35]. Other works present a small wearable device monitored remotely by a smartphone or tablet [36,37]. These approaches, although promising and important [38], still assume or favor a laboratory setting and/or special sensors and target an adult population. None of these approaches has studied the PA.

## Conclusions

We have now developed a non-contact automatic visual inspection method to analyze all three important parameters, particularly the PA. By using a portable, low-cost depth camera, this current method may be deployed in common clinical practice and greatly simplify the physician-patient interaction. We verified our results by comparing them to those of programmable lung simulators. In the future, we intend to verify them on recordings of many human patients by correlating the PA with readings from a conventional RIP belt device and the estimated TV with spirometer readings. In a preliminary in vivo testing scenario, we found a strong dichotomy in PA values between healthy and SMA patients. Overall, this technique offers the potential for improved patient comfort, continuous monitoring, and tailored treatment plans. Together with a growing amount of data, we intend to continue this research by incorporating machine learning methods for automatic detection of physical measurements and breath patterns correlated with expert medical evaluation, thus assisting physicians in their diagnosis and treatment.

## Figures and Tables

**Figure 9:**
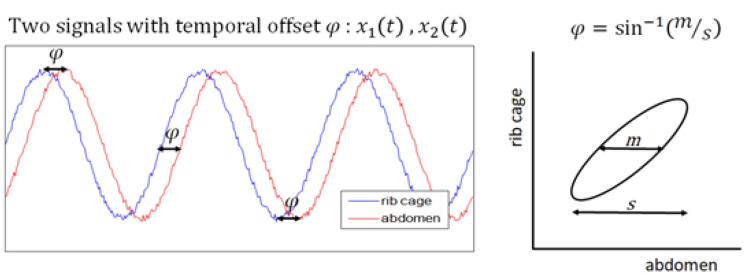
Phase shift between two sinusoidal signals (left) and the corresponding Lissajous curve (right), simulating an approximate 45° phase angle between rib cage and abdomen motions.

**Figure 10:**
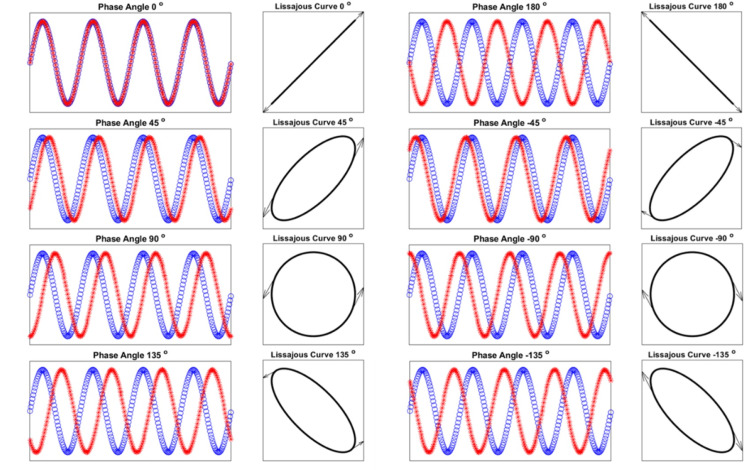
Phase shifted signals and corresponding Lissajous curves. Note the positive vs. negative angles (such as +45° representing a red signal slightly trailing a blue signal and -45° representing a red signal slightly preceding a blue signal), and also the clockwise vs. counterclockwise direction of the Lissajous curves.

## Appendices

Appendix 1

We calculated the PA using three different approaches: (1) a direct angle-between-vectors approach [39], (2) an approach based on the slant and direction of a Lissajous curve associated with two temporal signals, often presented in the literature [11,33,40,41], and (3) a Fast Fourier Transform (FFT)-based approach, calculating the angle between two complex numbers representing the Fourier transform of the dominant frequency of the signals.

The first approach is simply based on calculating the angle between vectors in n-dimensional real space, as defined in Equation (1). If we examine two signals with a temporal offset τ, x1(t) and x2(t-τ), sampled at n points in time, then the PA ϕ between them will be,

(1) $\phi = \arccos \left( \frac{\sum_{i=1}^n x_{1i} x_{2i}}{\sqrt{\sum_{i=1}^n x_{1i}^2 \sum_{i=1}^n x_{2i}^2}} \right)$

It is applicable if the two signals closely resemble smooth sine waves with fairly equal sampling. Although the signals can be seen as wave functions, they are noisy and clearly not sinusoidal; therefore, we decided to reject this approach. Lissajous curves are a general method for plotting two temporal signals, x(t) and y(t), on an xy planar graph. The angle is calculated as the arcsin of the ratio between half the rib cage excursion and the maximal abdomen excursion, as depicted in Figure 9. Several samples of time-shifted sinusoidal signals and their corresponding Lissajous curves are presented in Figure 10. This method works well for normal, consistent breathing, as in the case of adults, but may be problematic in babies and small children, where the breath cycles are not consistent, and the child exhibits movements independent of breath, thus producing noisy movement signals. Problems associated with the Lissajous curve method have already been pointed out by Chen and Hsaio [42].

The Fourier transform approach involves the following three steps: applying the FFT to the two signals, extracting the dominant frequency (the one yielding the highest amplitude), and calculating the angle between the two complex numbers representing the transform (the difference between the arctan of the ratio of the imaginary and real parts of these two complex numbers). According to this approach, when two signals have a temporal offset τ, x1(t) and x2(t-τ), the PA ϕ between them can be defined by the following formulation: let y1 and y2 denote the Fourier transform F of x1 and x2, respectively, as shown in Equation (2),

(2) $y_1 = F(x_1); \quad y_2 = F(x_2)$

and let ωmax denote the dominant frequency amongst the transformed signals, see Equation (3),

(3) $\omega_{\text{max}} = \arg_{\omega \in f} \max \left\{ |y_1(f)|, |y_2(f)| \right\}$

then, the phase shift ϕ is defined as the angle between two vectors in the complex plane, as in Equation (4)

(4) $\varphi = \angle \left( y_2(\omega_{\text{max}}), y_1(\omega_{\text{max}}) \right)$

or, in an equivalent explicit manner, as in Equation (5), where,

(5) $\Phi=arctan(&real;(y2(&omega;max))&image;(y2(&omega;max)))&minus;arctan(&real;(y1(&omega;max))&image;(y1(&omega;max)))$

We believe that this approach is better suited in the general case, handling noisy signals and inconsistent breath patterns. An advanced discussion of applying the Fourier transform for PA estimation was presented by Goldberg and Bokor [43].
